# Antibody-Based and Other Novel Agents in Adult B-Cell Acute Lymphoblastic Leukemia

**DOI:** 10.3390/cancers17050779

**Published:** 2025-02-25

**Authors:** Clifford M. Csizmar, Mark R. Litzow, Antoine N. Saliba

**Affiliations:** Division of Hematology, Department of Medicine, Mayo Clinic, Rochester, MN 55905, USA; litzow.mark@mayo.edu

**Keywords:** B-cell acute lymphoblastic leukemia, immunotherapy, blinatumomab, inotuzumab ozogamicin, CAR T-cell therapy, targeted therapies

## Abstract

Despite notable progress in managing B-cell acute lymphoblastic leukemia (B-ALL) over recent decades, particularly in pediatric cohorts where the 5-year overall survival (OS) reaches 90%, outcomes for the 10–15% with relapsed and refractory disease remain unfavorable. This disparity is further accentuated in adults, where individuals over the age of 40 years undergoing aggressive multiagent chemotherapy continue to have lower survival rates. While the adoption of pediatric-inspired treatment protocols has enhanced complete remission (CR) rates among younger adults, 20–30% of these patients experience relapse, resulting in a subsequent 5-year OS rate of 40–50%. For relapsed B-ALL in adults, there is no universally accepted standard salvage therapy, and the median OS is short. The cornerstone of B-ALL treatment continues to be the utilization of combined cytotoxic chemotherapy regimens to maximize early and durable disease control. In this manuscript, we go beyond the multiagent chemotherapy medications developed prior to the 1980s and focus on the incorporation of antibody-based therapy for B-ALL with an eye on existing and upcoming approved indications for blinatumomab, inotuzumab ozogamicin, other monoclonal antibodies, and chimeric antigen receptor (CAR) T cell products in frontline and relapsed/refractory settings. In addition, we discuss emerging investigational therapies that harness the therapeutic vulnerabilities of the disease through targeting apoptosis, modifying epigenetics, and inhibiting the mTOR pathway.

## Simple Summary

Despite high remission and survival rates in younger adults with B-cell acute lymphoblastic leukemia (B-ALL), managing relapsed disease across all age groups and optimizing treatment for older patients in both frontline and relapsed settings remain significant challenges. This manuscript explores advancements beyond conventional chemotherapy, focusing on antibody-based therapies such as blinatumomab and inotuzumab ozogamicin, CAR T-cell therapy, and emerging strategies targeting apoptosis, epigenetics, and the mTOR pathway to exploit therapeutic vulnerabilities in B-ALL.

## 1. Introduction

**Figure 1:**
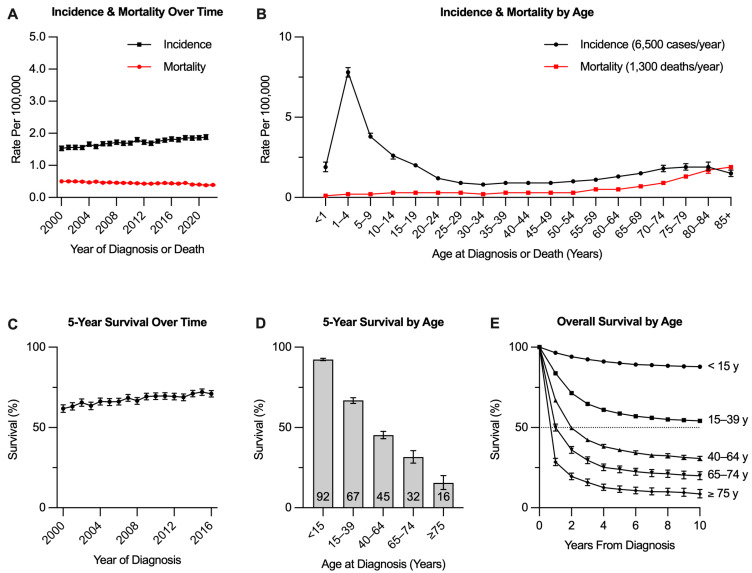
Epidemiology of acute lymphoblastic leukemia (ALL) in the United States. (**A**) Incidence and mortality of ALL by the year of diagnosis or death, respectively. (**B**) Incidence and mortality of ALL by the age at diagnosis or death, respectively; annualized incidence and mortality values are for all ages combined. (**C**) Five-year relative survival for ALL by year of diagnosis. (**D**) Five-year relative survival for ALL by age at diagnosis. (**E**) Overall survival of ALL by age at diagnosis. All data include both B/T cell lineage ALL and are from the National Cancer Institute (NCI) SEER*Explorer accessed 7 September 2024.

Precursor B-cell acute lymphoblastic leukemia (B-ALL) is a heterogeneous disease characterized by the neoplastic accumulation of immature B lymphoblasts that are arrested in the early stages of lymphoid differentiation, undergo dysregulated proliferation, and ultimately replace the normal hematopoietic compartment [1,2]. In the United States (US), the annual incidence of ALL has slowly increased over time and was estimated at 1.9 cases per 100,000 persons in 2021, or roughly 6500 new cases per year (Figure 1) [3,4].

There is a bimodal age distribution, with the highest incidence occurring in children aged 1–4 years (7.8 per 100,000) and a second peak amongst adults aged 75–84 years (1.9 per 100,000) [3]. Accordingly, most cases (60%) are diagnosed before the age of 20 years [2], and ALL accounts for only 0.2% of all cancer diagnoses in adults aged ≥20 years [4].

These age differences underlie corresponding disparities in outcomes. Intensive multiagent chemotherapy has improved the 5-year overall survival (OS) rates to >90% in pediatric B-ALL cohorts (ages < 15 years). However, 5-year OS declines to 60–70% amongst adolescents and young adults (AYA, ages 15–39 years) and further to about 40% for adults aged ≥40 years [3,5]. Although the use of pediatric-inspired treatment regimens has improved outcomes for AYAs [6], survival has historically been poor for older adults who cannot safely receive such regimens [7]. This is especially true amongst elderly individuals aged ≥75 years who comprise the second incidence peak with a 5-year OS of only 16% [3]. Thus, effective treatment of B-ALL in the elderly poses a unique challenge. Primary refractory and relapsed disease remains a challenge across age groups, with a median OS of only 6–9 months for AYAs and adults [8,9].

Of note, the different age categories used to guide management vary between regions. For instance, while commonly used in the US, the age groups of 18-39 years and 40–70 years are not widely adopted in Europe, and patients are often treated with modified higher intensity regimens up to the age of 55 or 60 years. While many institutions in the US use more stringent age categories to determine eligibility for pediatric-inspired regimens, European protocols often extend the use of intensive regimens to older age groups, reflecting key regional differences in practice.

Novel immune-engaging therapies and targeted small molecules aim to change this paradigm by increasing response rates, improving OS, and reducing the high toxicity associated with intensive chemotherapy. Anti-CD20 antibodies, the CD19xCD3 bispecific T cell engager (BiTE) blinatumomab, and the anti-CD22 antibody–drug conjugate (ADC) inotuzumab ozogamicin (InO) have recently been incorporated into practice guidelines [5,10]. This review summarizes the data supporting these practice-changing agents and further highlights emerging therapies that aim to leverage the increasing understanding of B-ALL pathogenesis.

### Classification

Genomic studies over the past two decades have identified a multitude of leukemogenic drivers—including oncogene mutations [11,12,13], copy number alterations [14,15], structural rearrangements [16,17,18], epigenetic modifications [19], and dysregulated gene expression profiles [20,21]—and these features now define the genetic subtypes of ALL. This molecular heterogeneity is highlighted by the current 5th edition of the World Health Organization (WHO) classification of haematolymphoid tumors [22] and the International Consensus Classification (ICC) of acute leukemias [23], with both schemata emphasizing the underlying structural lesions.

Clinically, however, B-ALL is broadly grouped into three categories based on the underlying relationship to the Philadelphia chromosome (Ph) [24]. Ph-negative B-ALL, wherein translocations involving *BCR::ABL1* are not present, is the most common group in children and comprises 25–50% of cases in adults [25]. Ph-positive B-ALL, defined by the presence of the canonical *BCR::ABL1* rearrangement, t(9;22)(q34;q11.2), represents the most common genetic subtype in adults [26]. Although rare in children (incidence 2–5%), the incidence of t(9;22) increases steadily with age, reaching >50% in those older than 50 years [27]. In Ph-like B-ALL, genetic lesions other than t(9;22) have given rise to a gene expression phenotype resembling that of Ph-positive B-ALL cases [28,29,30]. The incidence in adults is ~25%, although the molecular definition and identification of such cases continue to evolve [13].

In addition to Ph-based categorization, clinicogenomic risk stratification is essential. Traditional prognostic factors for B-ALL include age >60 years (although this threshold is variable) and a white blood cell (WBC) count >30 × 10^9^/L [31]. Genetic risk factors vary by study and, to a degree, between pediatric and adult populations. Monosomy 7, low hypodiploidy, *KMT2A* rearrangements, and complex karyotypes are generally regarded as high-risk cytogenetic features in adults [32,33]. Although numerous molecular associations have been described, no universal molecular risk stratification schema exists yet [24]. After induction, however, the strongest prognostic factor is the presence of measurable residual disease (MRD) [34]. MRD has become an essential prognostic element and, as discussed below, a key factor when considering consolidation strategies or allogeneic transplantation. Recent guidelines detail the necessary studies to facilitate risk stratification and MRD determination [5,24].

## 2. Philadelphia Chromosome Negative B-ALL

In contrast to the *BCR::ABL1* rearrangement in Ph-positive disease, which can be targeted by contemporary tyrosine kinase inhibitors (TKIs), the absence of a well-defined molecular target in Ph-negative disease has led to intense efforts to develop immunotherapies for this subset of B-ALL. Thus, antibody-based therapies such as rituximab, ofatumumab, blinatumomab, and InO have been most thoroughly evaluated in Ph-negative B-ALL.

### 2.1. Frontline Therapy

#### 2.1.1. Anti-CD20 Antibodies

Following the advent of rituximab, retrospective studies identified CD20 expression on ≥20% of leukemic blasts as an adverse prognostic feature independent of age, induction therapy intensity, or Ph chromosome status [35]. Specifically, CD20-positivity conferred a shorter duration of complete remission (CR) and inferior OS compared to CD20-negative cases. Thus, rituximab became one of the first immunotherapies to be explored in B-ALL (Table 1). In an early phase II study, the addition of rituximab to a modified regimen of hyper-fractionated cyclophosphamide, vincristine, doxorubicin, and dexamethasone (hyper-CVAD) improved the 3-year CR duration to 67% vs. 40% (*p* = 0.002) for those who did not receive rituximab, and the rate of relapse declined to 37% from 60%, respectively [36]. However, these benefits were restricted to patients younger than 60 years of age.

The phase III GRAALL-2005 study randomized patients aged 18–59 years with Ph-negative B-ALL to receive standard-of-care (SOC) chemotherapy with or without rituximab during all phases of therapy, including induction, consolidation, and maintenance [37]. Although there was a significant improvement in 4-year event-free survival (EFS) with rituximab (55% vs. 43%, *p* = 0.04) and the cumulative incidence of relapse (25% vs. 41%, *p* = 0.02), these benefits did not translate to improved OS (61% vs. 50%, *p* = 0.10), similar to the phase II study. However, more patients treated with rituximab were successfully bridged to allogeneic hematopoietic cell transplantation (HCT, 34% vs. 20%), and post-hoc analysis with outcomes censored at the time of HCT did suggest an OS benefit with rituximab (hazard ratio [HR] 0.55, *p* = 0.02). Adverse effects were not different between groups and, notably, the incidence of allergic reactions to asparaginase was lower in the rituximab arm (2% vs. 11%, *p* = 0.002). Thus, these data established the use of rituximab in combination with intensive chemotherapy as the standard of care for CD20+ B-ALL. 

The UKALL14 study examined the use of rituximab in combination with chemotherapy in patients aged 19–65 years with B-ALL irrespective of Ph-chromosome status or CD20 expression [38]. Unlike the GRAALL study, however, only four doses of rituximab were administered during the induction phase. EFS was not improved with four doses of rituximab, highlighting the need for extended anti-CD20 antibody therapy. The optimal dosing strategy and patient selection criteria for rituximab have not been fully defined but are unlikely to be major areas of investigation in the near future.

Studies utilizing ofatumumab instead of rituximab have yielded comparable results. A single-arm, phase II study in Ph-negative B-ALL combined hyper-CVAD with eight doses of ofatumumab throughout induction and consolidation [39]. CR rates after two cycles were 98% with 4-year relapse-free survival (RFS) and OS of 59% and 68%, respectively. A follow-up retrospective propensity score analysis (PSA) compared these patients to a historic cohort of Ph-negative B-ALL patients treated with rituximab and hyper-CVAD [50]. CR rates were similar with ofatumumab and rituximab (93% vs. 97%), and although there was a trend toward improved 4-year EFS (61% vs. 43%) and OS (66% vs. 48%), neither difference was significant. To date, ofatumumab and rituximab have not been directly compared in a prospective randomized fashion, and ofatumumab is seldom used in practice.

#### 2.1.2. Blinatumomab

Blinatumomab is a CD19xCD3 BiTE that has demonstrated remarkable efficacy across multiple phases of B-ALL therapy. Although initially investigated in relapsed and refractory (R/R) disease (see below), blinatumomab has since moved into earlier lines of therapy, with the addition of EMA- and FDA-approved indications as consolidation therapy for MRD-positive B-ALL in 2018 and as consolidation therapy irrespective of MRD in 2024. Meanwhile, several studies have incorporated blinatumomab into frontline induction regimens, particularly for older adults who cannot tolerate intensive chemotherapy.

For instance, the phase 2 SWOG 1318 trial showed that induction followed by consolidation with blinatumomab and POMP (prednisone, vincristine, methotrexate, and 6-mercaptopurine) maintenance was both well-tolerated and efficacious amongst adults aged 65 years and older (*n* = 29), inducing a CR in 66% and providing 3-year RFS and OS rates of 37% each [40]. Moreover, 92% of evaluable patients achieved MRD negativity by flow cytometry.

Meanwhile, preliminary data from the phase 2 German Multicenter Study Group for Adult ALL (GMALL) Bold study combining blinatumomab with abbreviated, dose-reduced induction chemotherapy in adults aged 55 years and older showed more encouraging results. Amongst the 50 patients accrued thus far, the CR rate was 76% with a 3-year OS of 65% [41,51]. The authors compared these outcomes to those receiving SOC therapy per the GMALL registry trial [52], and although such cross-trial comparisons should be interpreted with extreme caution, there appear to be favorable improvements in post-induction CR rates (85% vs. 78%), molecular CR rates (82% vs. 55%), and 3-year OS (67% vs. 49%) for patients treated with the Bold protocol.

Even amongst younger patients, there appears to be a benefit to early incorporation of blinatumomab. The phase 2 GIMEMA LAL2317 study added two doses of blinatumomab to cycles 3 and 6 of pediatric-inspired SOC induction chemotherapy for adults up to age 65 years [42,53]. After induction, the CR rate was 88%, and, after the first cycle of blinatumomab, 93% of patients were MRD-negative. The disease-free survival (DFS) and OS were 66% and 71%, respectively, after 3 years.

#### 2.1.3. Inotuzumab Ozogamicin

InO is an ADC composed of a humanized CD22-targeted antibody coupled to the calicheamicin derivative, ozogamicin [54]. CD22 is expressed on over 90% of leukemia blasts in B-ALL [55,56] and has since proven to be an effective immunotherapeutic target. Meanwhile, ozogamicin binds into the minor groove of DNA where it generates a di-radical, abstracts hydrogens from the DNA phosphodiester backbone, and ultimately causes double-strand DNA scission leading to cell death via apoptosis [57]. Based on the results of the pivotal INO-VATE trial [58], InO was EMA- and FDA-approved for adult R/R B-ALL in 2017, with an expanded indication for pediatric patients added in 2024. As with blinatumomab, however, InO has been swiftly evaluated as frontline therapy, again with an emphasis on older adults.

An early phase 2 study combined dose-reduced chemotherapy with four doses of InO (one dose on day 3 of each of the first four cycles) [43]. Amongst 52 patients aged 60 years or older, the CR rate was 85% with a 3-year OS of 56%. However, there was considerable toxicity, with 79% of patients experiencing grade 3 (or higher) infections. All patients experienced some degree of hepatotoxicity, including four cases (8%) of sinusoidal obstruction syndrome (SOS). Treatment-related mortality (TRM) occurred in six patients (12%), five from sepsis and one from SOS.

A similar phase 2 study added eight doses of InO to the first four cycles of a mini-hyper-CVD-based induction regimen for adults aged 60 years or older [44]. With this approach, the authors report a CR rate of 98%. SOS was observed in 5 of 58 patients (9%) with only three (5%) deaths in CR within the first 3 months. Concerningly, late deaths in CR from infection (*n* = 2) or secondary myeloid neoplasms (*n* = 2) were also reported. Still, these results compared favorably to a propensity-matched historical cohort of older patients who received traditional hyper-CVAD-based therapy. However, multiple protocol amendments, including the addition of blinatumomab to the consolidation and maintenance phases of a subset of patients (14%) confound the interpretation of these results.

The phase 2 EWALL-INO study offers a more generalizable evaluation of InO plus dose-reduced chemotherapy in this patient population (adults 55 years or older) [45]. Patients received up to five doses of InO across two cycles of induction chemotherapy followed by POMP maintenance. After both induction cycles, the CR rate was 90%, and the regimen offered 2-year leukemia-free survival (LFS) and OS rates of 50% and 54%, respectively, similar to the results of the above MD Anderson Cancer Center (MDACC) studies. MRD-negative status was achieved in 91% of patients. SOS was observed in three cases (2%).

Taking this approach further, the INITIAL-1 study omitted induction chemotherapy entirely, combining InO with dexamethasone alone in adults 55 years and older [46]. Although patients were offered up to three cycles of InO plus dexamethasone, all patients achieved a CR after two such cycles, and 71% achieved MRD negativity after the third cycle. When followed by up to six cycles of age-adapted GMALL consolidation and reinduction, the 3-year EFS and OS were 55% and 73%, respectively. No deaths were observed within the first 6 months, and only one case (2%) of SOS was observed.

InO has also been added to the pediatric-inspired CALGB 10403 regimen in AYA with newly diagnosed Ph-negative B-ALL. In the Alliance A041501 study, AYA (median age 27 years) were randomized to receive CALGB 10403 chemotherapy with (*n* = 111) or without (*n* = 116) two cycles of InO [47]. Neither the respective 3-year EFS (69% vs. 67%) nor OS (79% vs. 80%) were significantly improved. An increased incidence of grade 5 toxicities, predominantly infections, resulted in premature suspension of the study.

Collectively, these studies highlight the promise of incorporating InO into the frontline treatment of older adults with B-ALL, offering a means to reduce the intensity of chemotherapy (or eliminate it entirely), reduce toxicity, and improve survival. However, the risks appear to outweigh the benefits in younger patients receiving intensive chemotherapy, and larger prospective randomized studies using optimized InO dosing strategies are needed to formally evaluate these approaches.

#### 2.1.4. Blinatumomab Plus Inotuzumab Ozogamicin

Building upon these results, groups have attempted to sequence InO and blinatumomab to improve outcomes in older adults. A phase 2 study of reduced-intensity chemotherapy (mini-hyper-CVD) combined with InO during induction, followed by POMP maintenance and blinatumomab, demonstrated an encouraging CR rate of 89% with 5-year OS of 46% among adults aged 60 years and older [48]. Protocol amendments (to mitigate InO toxicity) limit the generalizability of this study.

Preliminary results of the Alliance A041703 study evaluating the chemotherapy-free regimen of InO induction followed by blinatumomab consolidation in older adults have been reported [49]. Among 33 patients aged 60 years or older, the CR rate was 97% with 1-year EFS and OS rates of 75% and 84%, respectively. Results with longer follow-ups are awaited to determine whether the omission of chemotherapy entirely is a reasonable option in the frontline setting.

### 2.2. Measurable Residual Disease

The presence of MRD after intensive-phase therapy confers a higher risk of relapse, shorter duration of response, and inferior survival [59]. Thus, great emphasis has been placed on identifying therapies capable of eliminating MRD, with blinatumomab showing exceptional promise (Table 2).

#### 2.2.1. Blinatumomab

In an early phase 2 study of 20 evaluable patients who remained MRD-positive after induction and consolidation, 16 (80%) became MRD-negative after treatment with blinatumomab [64]. After a median follow-up of 33 months, the RFS was 61%, and nine responders (56%) proceeded to HCT [65]. Ten patients (50%) remained in remission after 5 years, including five of those who did and did not undergo HCT [60].

Similar results were obtained in a smaller, separate phase 2 study which included nearly half of Ph-positive cases (who also received tyrosine kinase inhibitors [TKIs]) [62]. In this mixed population, MRD-negativity was achieved in 73% with estimated 3-year RFS and OS rates of 63% and 67%, respectively.

The larger phase 2 BLAST study of 113 patients recapitulated these results, with an MRD-negative CR rate of 78% and an estimated 18-month RFS rate of 54% [66]. After a median follow-up of 5 years, OS at 18 months was 67% with a median OS at 37 months amongst the cohort. Those who achieved MRD negativity in response to blinatumomab had superior OS to those who did not (median not reached vs. 14 months). Post-hoc analysis indicated that this benefit was maintained independent of HCT, suggesting that blinatumomab consolidation may be curative in a subset of patients [61]. These results led to the accelerated FDA approval (2018) of blinatumomab for patients with B-ALL in the first or second CR with MRD. This MRD-guided, risk-adapted approach is being further evaluated in the ongoing phase 3 GMALL 08/2013 trial [67].

#### 2.2.2. Inotuzumab Ozogamicin

InO has also been assessed in the MRD-positive population in a small phase 2 study of 26 patients, 16 (62%) of whom had Ph-positive disease [63]. After a median of three cycles (range 1–6), MRD negativity was achieved in 69%. SOS was noted in two patients (8%). Thus, while apparently less efficacious than blinatumomab in this setting, InO may be an alternative option for those who cannot receive the former.

### 2.3. Consolidation

Building on the prior studies demonstrating the efficacy of blinatumomab in inducing MRD-negative remissions, more recent studies have evaluated incorporating this agent into frontline consolidation regimens (Table 3).

In a phase 2 study, 38 adult patients with Ph-negative B-ALL were treated with up to four cycles of hyper-CVAD alternating with high-dose methotrexate and cytarabine; this was followed by four cycles of continuous blinatumomab consolidation [68]. Maintenance consisted of POMP with an additional dose of blinatumomab every fourth cycle. Notably, while only 25 of 29 (86%) evaluable patients were MRD negative prior to the first cycle of blinatumomab, all patients (100%) achieved MRD negativity after the first consolidation dose. The 3-year RFS was 73% and OS was 81%; however, no patient relapsed beyond 2 years from the start of therapy, highlighting the depth and durability of response.

The QUEST study is a phase 2 sub-study amendment of the larger GRAALL-2014/B trial, and preliminary data have been reported in abstract form for high-risk patients (defined as *KMT2A*-rearranged, *IKZF1*-deleted, and/or post-induction MRD-positive) [69]. After GRAALL-style induction therapy, 94 eligible patients went on to receive up to five cycles of blinatumomab as consolidation and maintenance. Unlike the prior MDACC study where all patients achieved MRD-negative status after one cycle of blinatumomab, only 72% of these high-risk patients achieved the same. Still, the 2.5-year RFS and OS rates were quite similar, at 72% and 79%, respectively.

The most compelling evidence supporting the use of blinatumomab as consolidation comes from the recently reported E1910 study [70]. Patients with Ph-negative B-ALL who had achieved an MRD-negative CR were randomly assigned to receive consolidation with four cycles of blinatumomab plus multi-agent chemotherapy or chemotherapy alone (*n* = 112 each). After a median follow-up of 43 months, the trial showed a superior 3-year RFS and OS in the blinatumomab arm, at 80% vs. 64% and 85% vs. 68% (*p* = 0.002), respectively. This OS benefit was seen in patients both younger and older than 55 years, although the absolute benefit was larger in the younger subgroup (95% and 70%, respectively). The results of E1910 led to the recent (2024) FDA approval of blinatumomab as consolidation for all patients with CD19-positive Ph-negative B-ALL irrespective of MRD status.

Finally, real-world data suggest that blinatumomab consolidation remains an effective strategy even after relapse and re-induction. In a pragmatic study of 31 patients with B-ALL in second CR, a complete MRD response was achieved in 86% of patients after one cycle of blinatumomab, and 45% of the patients in CR2 were ultimately bridged to HCT. The 3-year DFS and OS rates were 67% and 71%, respectively [71].

### 2.4. Relapsed Disease

As is often the case, blinatumomab, InO, and other agents were first evaluated in the R/R setting where, historically, outcomes were poor. Thus, the most mature data for these agents are in subsequent lines of therapy (Table 4).

#### 2.4.1. Epratuzumab

Prior to the successes of blinatumomab and InO (discussed below), the anti-CD22 monoclonal antibody, epratuzumab, was evaluated as salvage therapy for R/R B-ALL. However, these results were cumulatively disappointing. For example, the phase 2 SWOG S0910 study combined clofarabine, cytarabine, and epratuzumab to produce response rates of 52%, which were higher than previously reported for the combination of clofarabine and cytarabine alone (17%); median OS was 5 months [72]. Despite attempts to intensify therapy in younger patients, the addition of epratuzumab to hyper-CVAD did not appear more efficacious, with a CR rate of 33% and a median OS of only 3 months [73]. Development of epratuzumab has since ceased.

#### 2.4.2. Blinatumomab

Phase 2 studies of single-agent blinatumomab demonstrated encouraging efficacy, with CR rates ranging from 43–69% and median OS of 6–10 months [74,75]. In the larger confirmatory study, 40% of those who achieved CR proceeded to HCT. A follow-up analysis of the patients aged 65 years or older who were treated in these trials (*n* = 36) showed similar benefits, with a CR rate of 56%, three of whom (15%) proceeded to HCT [83]. Median OS was also 6 months in this age group. These data led to the first accelerated approval of blinatumomab by the FDA for R/R B-ALL in 2014.

Confirmatory data came from the landmark TOWER study, a phase 3 trial that randomized patients with R/R B-ALL to receive either blinatumomab (*n* = 271) or SOC chemotherapy (*n* = 134) [76]. CR rates were higher (44% vs. 25%, *p* < 0.001) and median OS was significantly improved (8 vs. 4 months, *p* = 0.01) with single-agent blinatumomab. MRD negativity was higher with blinatumomab (76% vs. 48%), and 24% of patients in each arm proceeded to HCT. Grade 3 or higher adverse event rates were similar between groups (87–92%), with fatal toxicities observed in 19% of patients receiving blinatumomab and 17% receiving chemotherapy. Neutropenia and infection were less common with blinatumomab whereas cytokine release syndrome was exclusively observed with BiTE therapy (5%); the rates of neurologic toxicity were similar between groups (8–9%). These data established blinatumomab as the standard of care for R/R CD19 + B-ALL and garnered regular FDA approval in 2017 for blinatumomab in this setting.

In the wake of the TOWER trial, subsequent studies have evaluated the efficacy of blinatumomab in later lines of therapy. Post-hoc analysis of TOWER data showed that, unsurprisingly, blinatumomab continued to offer higher rates of CR than chemotherapy even when used in second or later salvage (40% vs. 14%) [84]. Likewise, post-hoc analysis of patients who relapsed after HCT before enrolling in the phase 2 study revealed a 45% CR rate with 76% of these patients attaining MRD-negativity; median OS was similar to the overall study population at 9 months [85].

#### 2.4.3. Inotuzumab Ozogamicin

Early in development, single-agent InO was administered at a dose of 1.8 mg/m^2^ IV every 3–4 weeks to patients with R/R B-ALL [86]. This approach provided an overall CR rate of 57% and a modest median OS of 5 months. Four patients (8%) died of SOS. Thereafter, the administration schedule was modified to 0.8 mg/m^2^ on day 1 and 0.5 mg/m^2^ on days 8 and 15 of each 21–28 day cycle [78]. Although the CR rate was similar (59%), median OS was slightly better at 7 months. Most notably, however, SOS was observed in only one patient and was non-fatal, highlighting that an increased frequency of lower-dose InO maintains efficacy and improves safety.

These data formed the basis of the phase 3 INO-VATE ALL trial [58]. Adults (*n* = 218) aged 18 years or older (median 47 years) with R/R Ph-negative (85%) or Ph-positive (15%) B-ALL were randomized to receive either single-agent InO or investigator’s choice of three standard salvage chemotherapy regimens. The administration schedule of InO followed the hyperfractionated approach of the modified phase 2 regimen (above). The composite CR rate was 81%, much higher than observed in the phase 2 study, and was superior to the 29% rate achieved with chemotherapy (*p* < 0.001). More patients in the InO group achieved MRD negativity (78% vs. 28%), and median progression-free survival (PFS) was significantly improved with InO at 5 months vs. 2 months for chemotherapy (*p* < 0.001). After extended follow-up, the median OS was 8 vs. 7 months, respectively (*p* = 0.01) [87]. These data led to the EMA and FDA approval of InO for R/R CD22+ B-ALL in 2017.

An in-depth analysis of hepatic adverse events found treatment-emergent hepatoxicity of any grade in 51% of patients (*n* = 83 of 164) treated with InO, including 13% who developed SOS [88]. Importantly, SOS was more frequent among patients who underwent HCT (22%) compared to those who did not (3%). Five of these post-HCT SOS events were fatal. In multivariate analysis, both elevated pre-HCT bilirubin and a conditioning regimen containing two alkylating agents were associated with an increased risk of post-HCT SOS. Thus, alternate conditioning regimens should be strongly considered for patients treated with InO who are proceeding to HCT. In practice, InO is also limited to two cycles and prophylactic ursodiol is recommended for such patients.

Several other post-hoc analyses of the INO-VATE data have since been reported. Expectedly, patients who achieved MRD-negative CR (46% of treated patients) had the best survival outcomes, with a median OS of 14 months [89]. Also as expected, compared to younger patients, older patients (age 55 years and above) had a shorter median OS (6 vs. 9 months) despite similar remission rates [90]. Older patients were also more likely to experience SOS (41% vs. 17%). Additional analyses have reported improved outcomes compared to salvage chemotherapy irrespective of disease burden [91] and a longer time to subsequent salvage therapy for those who received InO [92]. Patient-reported outcomes were also more favorable with InO [93].

Following INO-VATE, a phase 2 study evaluated the combination of mini-hyper-CVD with InO as salvage therapy [79]. In contrast to INO-VATE, InO was administered at the higher 1.3–1.8 mg/m^2^ dose on day 3 of the first four courses of chemotherapy. This combination produced a CR rate akin to the initial phase 2 study of InO alone at 59%. The median RFS and OS, however, were encouraging at 8 and 11 months, respectively. SOS occurred in nine patients (15%), all of whom had undergone HCT.

Recently, InO has also been combined with dose-adjusted etoposide, prednisone, vincristine, cyclophosphamide, and doxorubicin (DA-EPOCH) for R/R disease [80]. The morphologic CR rate was an impressive 84%, with 88% being MRD-negative responses. Median EFS and OS were 10 and 17 months, respectively. SOS was reported in one (4%) case after subsequent HCT.

#### 2.4.4. Blinatumomab Plus Inotuzumab Ozogamicin

As blinatumomab was further developed and its potential for inducing MRD-negative remissions was realized, strategies sequencing InO and blinatumomab were readily explored. For instance, the aforementioned trial of mini-hyper-CVD plus InO [79] was amended to reduce and fractionate the dose of InO (similar to the modified phase 2 approach above), and four cycles of consolidation therapy with blinatumomab were added [94,95]. Amongst 43 patients treated after the protocol amendment, 30 of whom received blinatumomab, the CR rate was 67% with a median OS of 37 months [81]. Due to the smaller number of events, this OS was not significantly different from the median of 14 months reported for patients treated prior to the protocol amendment (*p* = 0.16). In the entire cohort, median OS was 17 months with a median RFS of 13 months amongst those who responded. Among responders, survival was not improved by HCT (47 vs. 37 months for those who did and did not undergo HCT, respectively, *p* = 0.98). Thus, the authors suggest that consolidation with blinatumomab may offer as much benefit as HCT when used as salvage therapy, although this hypothesis was not formally tested as a minority of patients in the study actually received blinatumomab.

To address this concern, the same authors retrospectively analyzed 21 patients treated with mini-hyper-CVD plus InO followed by blinatumomab in the study, eight (38%) of whom had R/R disease [82]. Five patients (63%) achieved a CR, all of whom were MRD-negative and proceeded to HCT. Still, the median OS for the R/R cohort was merely 6 months for these heavily pre-treated patients. Another retrospective analysis of patients treated with sequential InO and blinatumomab (*n* = 14, median age 41 years, age range 22–64 years) reported a median OS of 5 months following receipt of blinatumomab [96].

### 2.5. Biomarkers of Response and Resistance to Blinatumomab or InO

Correlative studies from the clinical trials of blinatumomab and InO have identified several biomarkers associated with response to these agents. Analysis of samples from the TOWER study demonstrated improved survival with higher pre-treatment platelet counts [97]. Lower disease burden was prognostic for a hematologic response, while increased frequencies of peripheral CD3+ T cells conferred an increased likelihood of achieving MRD-negativity. High response rates have also been reported amongst *CRLF2*-rearranged Ph-like ALL cases [98]. Conversely, loss of CD19 expression, CD19 mutation, and the presence of extramedullary disease have been associated with resistance to blinatumomab [98,99].

Similar clinical features have been reported for InO, with higher platelet counts being associated with superior response rates [87,100]. This study also reported inferior survival for patients with adverse cytogenetics (including complex karyotypes, t(4;11), t(9;22), and abnormal chromosome 17) and disease beyond first salvage. Conversely, genomic analysis of INO-VATE samples identified increased response rates with InO as compared to chemotherapy across leukemic subtypes, including those with Ph-like, *TP53*-altered, low-hypodiploid, and *KMT2A*-rearranged disease [101]. These data highlight that while InO offers superior response rates compared to chemotherapy, survival remains inferior for these high-risk patients compared to their molecularly and/or cytogenetically favorable counterparts.

### 2.6. Perspective

**Figure 2:**
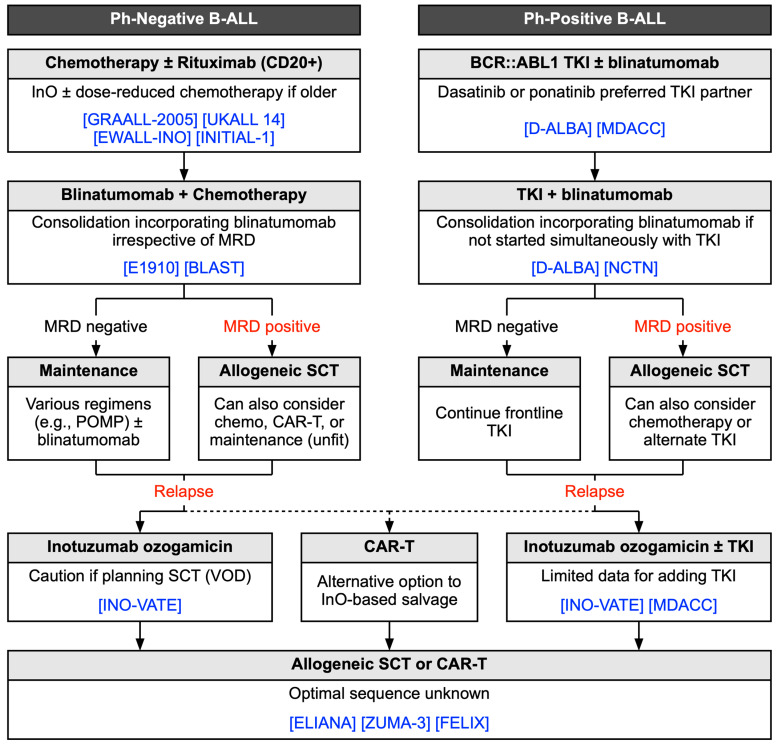
Emerging treatment approaches for Philadelphia chromosome (Ph)-positive and Ph-negative B cell acute lymphoblastic leukemia (B-ALL). Approaches represent one possible interpretation of the existing literature and are not intended to replace current treatment guidelines. Clinical trials upon which the proposed algorithms are based are noted in the blue brackets and are referenced in the text and corresponding tables.

Blinatumomab and InO have changed the paradigm of therapy for patients with Ph-negative B-ALL. The most practice-changing data in the frontline setting come from the E1910 study, establishing the use of blinatumomab as consolidation therapy for all-comers as opposed to only those with MRD [70]. Thus, a quickly evolving SOC is now multi-agent pediatric-inspired chemotherapy (plus at least eight doses of rituximab for CD20+ disease) with blinatumomab-based consolidation followed by maintenance and/or allogeneic HCT depending upon risk factors such as age, comorbidities, genomic features, and MRD status (Figure 2).

This approach may be further tailored for older adults who cannot tolerate pediatric-inspired chemotherapy regimens, where the (off-label) use of InO plus dose-attenuated chemotherapy (e.g., mini-hyper-CVD) is an attractive alternative to intensive induction [48]. Notably, this earlier use of blinatumomab as universal consolidation somewhat obviates the prior application as a risk-adapted therapy for MRD-positive disease, and persistent MRD refractory to blinatumomab remains a clinical challenge, highlighting a potential area where CAR T cell therapy could make a meaningful impact.

The approval of InO for relapsed disease also represents a significant improvement over historical approaches and is recommended by guidelines based on the INO-VATE trial [5,58]. Blinatumomab is also highly effective as salvage therapy, as shown in TOWER [76], but its role in relapsed settings is becoming less clear as more patients are treated with blinatumomab upfront as part of consolidation and maintenance. Thus, InO may be preferable for these patients who are less likely to have prior exposure to anti-CD22 therapy. Caution should be exercised when giving InO to patients who may proceed to allogeneic HCT, and dual-alkylator conditioning regimens should be avoided in those treated with InO.

## 3. Philadelphia Chromosome Positive B-ALL

Historically, Ph-positive B-ALL was characterized by primary resistance to chemotherapy and thus was associated with particularly poor outcomes [102,103,104]. Fortunately, the advent of TKIs targeting BCR::ABL1 dramatically improved survival and now forms the foundation of therapy for Ph-positive B-ALL (as recently reviewed elsewhere [26]). This section discusses the subsequent addition of immunotherapy to this field, highlighting recent opportunities for chemotherapy-free regimens.

### 3.1. Induction and Consolidation

Induction therapy with a TKI plus non-intensive chemotherapy is the standard of care for patients with Ph-positive B-ALL, based on the GRAALL-2005 study which demonstrated that imatinib combined with reduced-intensity chemotherapy produced a higher CR rate (98% vs. 91%), lower treatment-related mortality (day 60 mortality rates of 2% vs. 9%), and similar OS (median 4.1 vs. 3.3 years) as compared to the combination with intensive hyper-CVAD [5,105]. A pediatric phase 3 study from China [106] and an adult US intergroup study ushered in dasatinib as a likely superior alternative to the first-generation TKI imatinib and led to the FDA approval of dasatinib for Ph-positive ALL in 2009 [107]. The recent PhALLCON trial, wherein patients were randomized to receive European Working Group on Adult ALL (EWALL)-based low-intensity chemotherapy with either imatinib or ponatinib, showed even further improvement with the third-generation TKI, ponatinib [108]. The MRD-negative CR rates for those treated with ponatinib vs. imatinib were 34% vs. 17% (*p* = 0.002). Median PFS was also superior in the ponatinib arm (20 vs. 8 months), although survival data are still immature. Thus, the addition of immunotherapy to frontline regimens aims to improve MRD-negative response rates, extend the durability of remission, and/or reduce the toxicity of the already dose-reduced chemotherapy backbone (Table 5).

#### 3.1.1. Rituximab

Anti-CD20 antibody therapy has also been applied to Ph-positive B-ALL, although these data are largely derived from studies enrolling both Ph-negative and Ph-positive patients such as UKALL14 [38]. In that study, 86 (30%) patients in the rituximab plus chemotherapy group were Ph-positive and exploratory analysis suggested a trend toward benefit with rituximab (HR 0.77, *p* = 0.58), again with the limitation that only four doses of rituximab were administered. A single-arm, phase II study in Korea examining the addition of rituximab to SOC chemotherapy enrolled 32 patients with Ph-positive B-ALL [109]. Eight doses of rituximab were administered throughout induction and consolidation. The CR rate was 100% with 4-year RFS and OS each at 52%. Thus, while rituximab can be considered for CD20+ cases of Ph-positive B-ALL, the data are less robust than in Ph-negative disease [5].

#### 3.1.2. Blinatumomab

Perhaps the most striking role of blinatumomab in Ph-positive disease is its potential to replace the chemotherapy backbone entirely. The phase 2 D-ALBA study enrolled 63 patients with newly diagnosed Ph-positive B-ALL and administered induction therapy with dasatinib and dexamethasone alone followed by two or more cycles of blinatumomab as consolidation [113]. After the 85-day induction period, 98% of patients had a morphologic CR while 29% had a molecular response. Molecular responses increased to 60% after the second consolidative dose of blinatumomab, and further to 81% after four cycles. After long-term follow-up (median 53 months), the DFS and OS were 76% and 81%, respectively [110]. Notably, 29 patients never received chemotherapy or HCT, and 28 of those remained in CR at the last follow-up. HCT was pursued in 24 patients, 13 of whom had persistent MRD, and 20 remained in CR at the last follow-up. Those with *IKZF1* deletions plus co-existing deletions of *PAX5* or either *CDKN2A* or *CDKN2B* (collectively, *IKZF1^plus^*) had considerably worse outcomes, with <10% achieving a molecular response, and shorter survival (DFS 46% and OS 50% at 48 months).

A second phase 2 study (SWOG 1318), which enrolled concurrently with D-ALBA, evaluated a similar approach in patients aged 65 years and older with Ph-positive B-ALL [111]. Patients received dasatinib and prednisone induction followed by three cycles of blinatumomab consolidation. Of the 24 patients, 22 (92%) achieved a CR while the other two (8%) expired during induction therapy. Of 16 evaluable patients, six (38%) were MRD-negative by flow cytometry at day 28, and 17 of 19 (89%) achieved a molecular response at some point in therapy. DFS and OS were 77% and 87% at 3 years, respectively. After extended follow-up, the median OS was 6.5 years [114].

Whereas prior studies introduced blinatumomab later in treatment, a recent phase 2 study combined ponatinib and up to five cycles of simultaneous blinatumomab as initial therapy [112]. Amongst the 40 patients with newly diagnosed Ph-positive B-ALL treated, the CR rate was 93%, with 87% achieving a molecular response after the five cycles of blinatumomab. One-year estimates of EFS and OS were both 95%. Encouraging 3-year follow-up results were recently reported, with sustained EFS of 77% [115].

While these data strongly suggest that TKI/blinatumomab combinations can enable chemotherapy-free treatment for a subset of patients with Ph-positive B-ALL, each of the two described approaches are currently being evaluated in phase 3 trials (EA9181; NCT04530565 and GIMEMA ALL2820; NCT04722848). Moreover, such chemotherapy-free regimens have raised concern for a higher incidence of CNS relapse. Even with systemic chemotherapy regimens that incorporate CNS-penetrant doses of cytarabine and methotrexate (such as hyper-CVAD), multiple doses of dedicated IT chemotherapy are still required to mitigate this risk [116]. Thus, it remains to be seen whether additional measures—such as an increased number of IT administrations—will be necessary to safeguard against CNS relapse when the systemic agents are omitted.

### 3.2. Relapsed Disease

Most patients with Ph-positive B-ALL have developed TKI resistance mutations. Re-exposure to later-generation TKIs produces molecular response rates of less than 50% and median OS remains less than 1 year [117,118]. Thus, new strategies are needed to improve outcomes in this high-risk population (Table 6).

#### 3.2.1. Blinatumomab

As a single agent, blinatumomab was evaluated as second-line therapy for Ph-positive B-ALL in the phase 2 ALCANTARA study [122]. Amongst 45 adult patients treated with blinatumomab, 16 (36%) achieved a CR within the first two cycles, with 14 (88%) of whom becoming MRD-negative. Seven (44%) were bridged to HCT. After extended follow-up, the median RFS and OS were 7 and 9 months, respectively [119].

These patients were later compared to a historical SOC cohort (*n* = 55) in a PSA [123]. In the PSA model, both the CR rate (36% vs. 27%) and median OS (7 vs. 6 months) favored blinatumomab.

A post-hoc analysis examined the outcomes of Ph-positive patients treated with blinatumomab on the TOWER trial [124]. Amongst 142 patient samples available for RNA sequencing, 15 (11%) were found to harbor Ph-like alterations, including nine who were treated with blinatumomab and six who had received SOC therapy. CR rates were similar between Ph-like and Ph-negative patients treated with blinatumomab (44% vs. 46%, respectively), but not between those who received chemotherapy (0% vs. 14%). Median OS was also numerically similar between Ph-like and Ph-negative patients treated with blinatumomab (7.9 vs. 8.4 months), and while these two groups were not directly compared, outcomes were more favorable with blinatumomab vs. chemotherapy.

Early data have also evaluated blinatumomab in combination with a TKI for R/R disease. The same phase 2 study that evaluated concurrent ponatinib and blinatumomab in newly diagnosed patients also included 14 patients with R/R B-ALL [112]. Although the CR rate was similar to the untreated patients (92% vs. 96%, respectively), fewer patients achieved a molecular response (79% vs. 87%) and both 1-year EFS (57% vs. 95%) and OS (79% vs. 95%) were shorter. Median EFS was 15 months while median OS has not yet been reached.

A retrospective analysis of patients from 13 French centers who had received at least one cycle of simultaneous ponatinib plus blinatumomab reported encouraging results [125]. Amongst the 26 patients with a median age of 58 years (range 18–81 years), morphologic CR was achieved in 25 (96%) patients within three cycles of therapy, and 22 (89%) achieved a molecular CR. Eight patients proceeded to HCT. With a median follow-up of nearly 3 years, the median EFS was an impressive 15 months with a median OS of 20 months.

Although blinatumomab has not been compared to chemotherapy in a prospective randomized fashion for R/R Ph-positive and Ph-like B-ALL, these data collectively suggest that blinatumomab is preferable to chemotherapy for the treatment of relapsed disease in these subgroups. As with Ph-negative B-ALL, however, the increasing use of blinatumomab in the frontline setting may limit the generalizability of these studies.

#### 3.2.2. Inotuzumab

Data evaluating InO for R/R Ph-positive B-ALL are more limited. A phase 1/2 study administered single-agent InO to 16 patients for whom TKI therapy had failed [120,126]. The CR rate was 56%, with all responding patients achieving MRD negativity. Three (19%) patients underwent HCT. Median PFS and OS were 4 and 7 months, respectively. SOS was reported in two cases.

This same report also described the outcomes of Ph-positive B-ALL patients (*n* = 22) treated in the INO-VATE study [120]. CR rates were higher in this cohort at 73%, with 81% of responders achieving MRD negativity. Nine (41%) patients proceeded to HCT. Median PFS and OS were similar at 4 and 9 months, respectively. SOS was reported in an additional two cases, both occurring after HCT.

As with blinatumomab, there is interest in combining InO with TKI therapy. A phase 1/2 study of simultaneous bosutinib and InO therapy produced a CR rate in 15 of 18 patients treated (83%), with 11 of the responders (73%) achieving MRD negativity [121]. Complete molecular response was observed in 10 of 18 (56%). The median duration of response was 8 months with a median OS of 14 months. Six patients underwent HCT. No deaths were reported within 30 days of starting treatment, and no cases of SOS were reported after a median follow-up of 44 months.

These early studies suggest InO—either alone or with a TKI—may be an efficacious option for Ph-positive B-ALL as well. 

### 3.3. Perspective

As with Ph-negative disease, blinatumomab has the potential to dramatically change the way Ph-positive B-ALL is treated. Whereas the advent of BCR::ABL1 TKIs enabled the use of reduced-intensity chemotherapy [105], blinatumomab provides an avenue to eliminate it entirely from the frontline setting [110,112] (Figure 2). This is particularly enticing for older adults or those with comorbidities for whom even reduced-intensity chemotherapy may be challenging [111,127]. Moreover, the deep and durable remissions reported by these early studies may obviate the need for HCT in a subset of patients, reserving it for patients with persistent MRD after several cycles of blinatumomab. While these findings will need to be confirmed in prospective randomized trials, the existing data show considerable promise for such a chemotherapy-free approach.

## 4. Philadelphia Chromosome Like B-ALL

Ph-like B-ALL includes a heterogeneous group of high-risk cytogenetic and molecular alterations that give rise to a gene expression profile reminiscent of Ph-positive B-ALL [28,29,30]. Although there is no universal or all-encompassing definition of what constitutes Ph-like B-ALL, at least two distinct subgroups have emerged and are recognized by the ICC [23]: ABL class alterations and JAK-STAT pathway alterations. A third subgroup, often designated as “other”, captures those with RAS pathway alterations or rare kinase fusions (such as *NTRK3*, *FGFR1*, or *PTK2B*) [23,128].

Due to the heterogeneity of this group, there is no universally accepted standard of care [5]. By historical extrapolation, patients who are fit for intensive chemotherapy typically receive a multi-agent regimen consistent with their age group. More recently, there has been intense interest in incorporating TKI therapy for patients with targetable ABL-class (including *ABL1*, *ABL2*, *CSF1R*, *PDGFRA*, *PDGFRB*, and *KIT*) or JAK-STAT/CRLF2 (including *JAK2, EPOR*, and *CRLF2*) alterations. A substantial body of evidence supporting this approach is derived from retrospective and early-phase pediatric/AYA studies, where the addition of ABL-targeted TKIs led to improved rates of remission and relapse [129,130,131,132]. There are also early data supporting the use of ruxolitinib for select patients with *JAK2* or *EPOR* alterations [133]. Although there are approved inhibitors targeting FLT3, NTRK3, FGFR1, and various RAS alterations for other malignancies, there are no robust data regarding the use of agents for Ph-like ALL. Still, the improvements seen with ABL- and JAK-targeted TKIs are encouraging and highlight the importance of incorporating these agents when appropriate. These nuances and their supporting data are expertly reviewed elsewhere [128].

## 5. Investigational Agents

**Figure 3:**
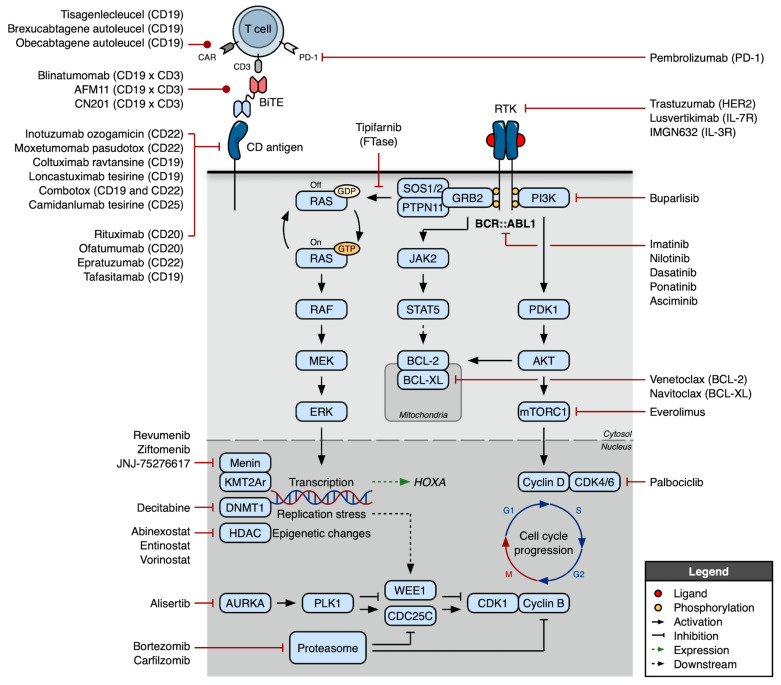
Approved and investigational agents for the treatment of B cell acute lymphoblastic leukemia (B-ALL). Numerous immunotherapies—including monoclonal antibodies, antibody–drug conjugates, bispecific T cell engagers (BiTEs), and immune checkpoint inhibitors—have been evaluated as treatments for B-ALL. With an increasing understanding of the biology of B cell leukemias, targeted small molecules have also been explored, the majority of which are being repurposed from other disease states rather than developed intentionally for B-ALL (the notable exception being BCR::ABL1 targeted tyrosine kinase inhibitors [TKIs]).

Despite the successes of blinatumomab and InO, their administration logistics, cost, toxicity, and less-than-optimal responses in R/R cases continue to motivate the development of additional agents for B-ALL (Figure 3).

While many agents have been evaluated in early-phase clinical trials of heavily pre-treated patients (Table 7), none apart from CAR T cells have yet matched the efficacy seen with blinatumomab or InO.

### 5.1. Drug Classes

Numerous immunotherapies and molecularly targeted agents have been evaluated in B-ALL. Antibody-based therapies have targeted both well-validated leukemia-associated antigens and non-traditional targets alike. Meanwhile, small molecules have largely been repurposed from other malignancies based on pre-clinical data suggesting potential therapeutic efficacy.

#### 5.1.1. Antibody-Based Therapies

Several additional agents targeting CD19 and CD22 have been developed. The Fc-engineered anti-CD19 antibody, tafasitamab, was evaluated in a phase 2 study, but CR rates were low (9%) and the duration of response was short (<2 months) [140]. Several ADCs targeting CD19, CD22, or both, have also been evaluated, but none have produced CR rates >20% and toxicities were considerable [134,135,136,137]. The tetravalent CD19xCD3 bispecific construct, AFM11, reported a CR rate of 21% in a phase 1 study, but the median OS remained short (2 months) in this high-risk population [138]. Phase 1 data from a new CD19xCD3 bispecific antibody, CN201, have been reported in abstract form with an encouraging CR rate of 32% with 75% of responders achieving MRD negativity; both rates improved with dose escalation [139].

Non-traditional (and less well-validated) antigens have also been explored. A CD123-targeted ADC, IMGN632, has been evaluated pre-clinically; CD123 expression was reported in 164 of 183 (90%) of primary B-ALL samples, and IMGN632 exhibited picomolar activity in vitro [160]. Likewise, a CD127 (interleukin 7 receptor [IL-7R]) was recently shown to be expressed in a majority of ALL cases, and a CD127-targeted antibody, lusvertikimab, showed potent activity in patient-derived xenograft models [161]. One patient with Ph-negative was treated on a phase I study of the anti-CD25 ADC, camidanlumab tesirine; they did not respond [162].

Given the remarkable efficacy of immune checkpoint blockade in other solid and hematologic malignancies, pembrolizumab was administered to 12 patients with MRD-positive R/R B- or T-ALL in a phase 2 study. Although well-tolerated, only one patient (who had B-ALL) experienced an MRD response which was brief (3 weeks); the trial was thus terminated [141]. As human epidermal growth factor receptor 2 (HER2) is upregulated on approximately one-third of B-ALL samples, a phase 2 study administered trastuzumab to 15 patients with HER2-positive R/R B-ALL; although treatment was well-tolerated, no CRs were observed [142]. Whether new HER2-targeted ADCs would provide additional efficacy remains to be tested.

#### 5.1.2. Menin Inhibitors

Menin inhibitors are a promising addition to the therapeutic armamentarium for acute leukemias that harbor a KMT2A (MLL) rearrangement. Approximately 10% of B-ALL cases harbor a *KMT2A* rearrangement. Recent studies have demonstrated the efficacy of menin inhibitors in preclinical and clinical settings. Revumenib is the first agent in this class to garner FDA approval in *KMT2A*-rearranged acute leukemia, including ALL, following The AUGMENT-101 study. Revumenib has not been approved by the EMA. AUGMENT-101 evaluated revumenib in R/R *KMT2A*-rearranged acute myeloid leukemia (AML, *n* = 49) and ALL (*n* = 7) and showed an overall response rate of 63% and composite CR rate of 23% [163]. Adverse events of note include differentiation syndrome and QTc interval prolongation, which require close monitoring. Preclinical studies have highlighted the potential of other menin inhibitors, such as MI-3454, which induced remission in mouse models (including patient-derived xenografts) of *KMT2A*-rearranged leukemia [164]. Furthermore, menin inhibitors have been found to work synergistically with other therapeutic agents. For example, combining menin inhibitors with DOT1L inhibitors has shown enhanced efficacy in *KMT2A*-rearranged ALL, suggesting a promising therapeutic strategy. Another study identified a significant synergy between menin inhibitors and proteasome inhibitors, such as carfilzomib, in infant B-ALL cells [165]. While the current focus of development for menin inhibitors is in AML, bleximinib (JNJ-75276617), enzomenib, and ziftomenib are also under investigation in ALL with *KMT2A* rearrangements [143,163,166,167,168].

#### 5.1.3. BH3-Mimetics

The pro-apoptotic BH3-mimetic, venetoclax, has revolutionized the treatment of acute myeloid leukemia (AML) and chronic lymphocytic leukemia (CLL). Thus, there has been intense interest in evaluating this class of compounds across hematologic malignancies, including B-ALL.

In an attempt to mitigate the dose-limiting thrombocytopenia associated with the mixed BCL-2/BCL-XL inhibitor, navitoclax, this agent was combined with both the selective BCL-2 inhibitor, venetoclax, and low-intensity vincristine-based chemotherapy in a phase 1 trial of R/R B-ALL and T-ALL across all ages [144]. Of 47 total patients treated, 13 (28%) achieved a CR, and the median time on therapy was 2 months. However, toxicity was considerable, and 10 (21%) patients experienced fatal treatment-emergent adverse effects. Navitoclax is no longer in development.

The combination of venetoclax with mini-hyper-CVD produced more favorable outcomes, with a CR rate of 57% and a median response duration of 6 months; the median OS was 7 months [145]. Two patients (9%) expired from treatment-related infections.

At least two studies (one retrospective) have also combined venetoclax with ponatinib to garner chemotherapy-free approaches for patients with R/R Ph-positive B-ALL [146,169]. These have produced encouraging CR rates of 80–90% of patients treated at therapeutic dose levels, including in patients with prior exposure to TKIs, blinatumomab, or HCT. Molecular CR rates were reportedly in 44–47% with a median OS of over 1 year in the retrospective analysis [169] and not reached in the prospective study [146].

Finally, a phase 1/2 study in China combined venetoclax with the investigational TKI, olverembatinib, for frontline treatment of Ph-positive B-ALL. All 10 patients achieved an MRD-negative CR (100%), with 90% also exhibiting a molecular CR. Follow-up was short with a median of 7 months, at which time no patient had relapsed or expired [147].

Collectively, these data highlight the promise of BCL-2 targeted therapy in combination with reduced-intensity chemotherapy or TKIs. Multiple phase 2 studies evaluating venetoclax in various combinations are currently enrolling at various centers internationally.

#### 5.1.4. Histone Deacetylase Inhibitors

Histone deacetylase (HDAC) inhibitors have long been evaluated in hematologic malignancies [170,171], with several garnering FDA approvals for T cell lymphomas. More recently, three HDAC inhibitors—vorinostat, abexinostat, and entinostat—have been evaluated in early-phase trials enrolling B-ALL patients. Monotherapy with abexinostat was not efficacious with no reported responses [149]. When combined with clofarabine, entinostat produced CRs in four of 28 patients (14%), all of whom had newly diagnosed disease; the median OS was 42 months for these few patients [150]. The addition of vorinostat and decitabine to chemotherapy produced a higher CR rate of 31% in R/R ALL patients, but survival remained short with a 2-month RFS of merely 45% [148]. More efficacious combination partners will be necessary to advance HDAC inhibitors in this space.

#### 5.1.5. PI3K-AKT-mTOR Pathway Inhibitors

Activation of the mechanistic target of rapamycin (mTOR) pathway contributes to the hyperproliferative phenotype of ALL, and recent genome-wide knockout screens have confirmed mTOR complex 1 (mTORC1) as a crucial mediator of glucocorticoid sensitivity in B-ALL [172]. Thus, inhibitors of the phosphoinositol-3-kinase (PI3K)-AKT-mTOR pathway are of particular interest. The combination of the canonical mTOR inhibitor, everolimus, with intensive hyper-CVAD chemotherapy produced a CR rate of 25% in a diverse population of R/R B-ALL and T-ALL patients; the median OS was about 6 months [151]. More encouraging results were obtained with everolimus in a pediatric cohort [173], whereas temsirolimus produced excessive toxicity when combined with UKALL R3-based re-induction therapy [174].

The mTORC1/2 inhibitor, sapanisertib, was recently evaluated as monotherapy in 15 heavily pre-treated patients with R/R B-ALL or T-ALL [152]. No patient achieved a CR and median OS was 2 months. Mucositis and cytopenias were common. Moving upstream in the pathway, the PI3K inhibitor, buparlisib, was also evaluated as monotherapy in a phase I trial of advanced leukemias that enrolled one patient each with ALL (B or T not specified) and mixed phenotype acute leukemia with no responses observed [153]. Collectively, these studies suggest that single-agent modulation of the PI3K-AKT-mTOR pathway is likely insufficient and that a synergistic therapy partner will be necessary to successfully intervene on these targets. Moreover, it remains to be determined whether there is a subtype of patients, such as those with *PAX5* alterations [12], that may benefit more from mTOR-targeted therapies.

#### 5.1.6. RAS Pathway Inhibitors

Mutations in *RAS* family genes are amongst the most frequently encountered genomic alterations in cancers and drive aggressive biology in many solid and hematologic malignancies [175,176]. Before the advent of RAS-specific inhibitors, attempts to inhibit RAS signaling relied upon upstream or downstream mediators of the pathway.

Membrane-bound farnesyltransferases localize RAS to the intracellular membrane to facilitate activation. In a phase 1 trial of tipifarnib in R/R AML or ALL, neither of the two ALL patients enrolled responded [154]. In a follow-up study, tipifarnib was combined with the proteasome inhibitor, bortezomib; however, the sole ALL patient in this trial also did not respond [155]. Tipifarnib was challenging to tolerate, with frequent dose-limiting toxicities.

Downstream of RAS, aurora kinase A (AURKA) both directly interacts with MYC and activates polo-like kinase 1 (PLK1), thereby promoting G2/M cell cycle progression [177,178,179]. Alisertib has since emerged as a candidate AURKA inhibitor, but monotherapy with alisertib failed to produce meaningful responses in a pediatric/AYA cohort of ALL patients [180].

Thus, while indirect inhibition of RAS-mediated signaling has not been efficacious, the recently approved direct KRAS inhibitors (and emerging pan-RAS inhibitors) have yet to be evaluated in ALL.

#### 5.1.7. Cyclin Dependent Kinase Inhibitors

Like RAS inhibitors, cyclin-dependent kinase (CDK) inhibitors modulate cell cycle progression and have emerged as important therapies in solid tumors, particularly hormone receptor-positive breast cancer. Palbociclib, a CDK4/6 inhibitor, was evaluated as monotherapy in R/R B-ALL [156]. One of seven (14%) patients enrolled achieved a morphologic CR with detectable MRD but relapsed after the second cycle of therapy. All patients had expired by the close of the trial.

#### 5.1.8. Proteasome Inhibitors

Proteasome inhibitors have become a mainstay of therapy for malignant dysproteinemias, but their utility in leukemias is limited [181]. In a phase 1 study of carfilzomib plus hyper-CVAD in newly diagnosed Ph-negative B-ALL and T-ALL patients, all 10 (100%) experienced a CR, and seven of the eight (88%) B-ALL patients in the study achieved MRD negativity [157]. No patient relapsed while on study therapy or during the 28-day follow-up period; however, survival was not reported. All patients experienced a grade 3 or 4 adverse events.

Bortezomib was added to rituximab and SOC intensive chemotherapy in a single-arm phase 2 study of newly diagnosed, Ph-negative, CD20+ B-ALL patients in India [158]. The CR rate was 85% with 68% MRD-negative after induction and improved to 88% after consolidation. EFS and OS were each 79% after 21 months. Most patients (88%) experience a grade 3 or 4 adverse events, with infection being the most common. One patient expired during induction therapy.

#### 5.1.9. DNA Methyltransferase Inhibitors

While DNA methyltransferase inhibitors such as decitabine and azacitidine (also colloquially known as hypomethylating agents) are a mainstay of therapy for high-grade myeloid neoplasms, they have found little use in lymphoid leukemias. Decitabine was evaluated alone or in combination with SOC hyper-CVAD in a phase 1 trial of R/R ALL, most of whom (82%) had B-ALL [159]. As a single agent, decitabine induced a CR in only 3% of patients; when combined with hyper-CVAD, however, CR rates increased to 28%, for an overall CR rate of 15%. Median OS for all patients was 3 months but was significantly longer amongst those who responded at 15 months. Notably, five patients treated on the decitabine + hyper-CVAD arm had either relapsed after prior response to hyper-CVAD (*n* = 2) or were refractory to hyper-CVAD (*n* = 3) and responded only after the addition of decitabine on the protocol. Treatment was generally well-tolerated without unexpected toxicities.

### 5.2. Perspective

Although most studies of these investigational agents are conducted in heavily pretreated patients who are inherently resistant to therapy, few agents have come close to recapitulating the early results that were seen with blinatumomab and InO in similar populations. Perhaps the most promising agents are the menin inhibitors for *KMT2A*-rearranged disease and the BH3 mimetics, such as venetoclax, which produced response rates >50% when combined with either dose-reduced chemotherapy (mini-hyper-CVD) or ponatinib [145,146,163]. While encouraging, these results should be interpreted with caution as these single-arm studies did not include a comparison arm to determine whether these response rates were truly due to the addition of venetoclax or the well-established partner therapy (as is the case for most early-phase studies). Thus, randomized prospective trials will be needed to determine whether BH3 mimetics will be as transformative for ALL as they have been for other hematologic malignancies. In the quest to find novel therapies in areas of unmet need, innovative approaches such as ex vivo drug sensitivity assays or pharmacologic profiling of patient-derived ALL cells may accelerate the identification of effective agents, especially in rarer disease subtypes [182,183,184,185,186].

## 6. Chimeric Antigen Receptor T Cells

Chimeric antigen receptor (CAR) T cell therapy has generated promising results in R/R B-ALL patients. To date, three CD19-targeted CAR T cell products have been FDA- and EMA-approved for ALL—tisagenlecleucel, brexucabtagene autoleucel, and obecabtagene autoleucel—based on the results of the ELIANA [187], ZUMA-3 [188], and FELIX [189] studies, respectively. However, the ELIANA and ZUMA-3 trials enrolled relatively young patients (median ages 11 and 40 years, respectively), and, although response rates were high (> 0%), these results may not be generalizable to many adult patients. Encouragingly, a recent meta-analysis of 38 studies enrolling 2,134 patients reported similarly high response rates (76%) with median EFS and OS of 13 and 36 months, respectively, with no significant survival differences when patients were stratified by age range (pediatric/AYA vs. adult) [190]. Otherwise, the approvals of blinatumomab and InO have raised questions regarding the comparative efficacy of these therapies (especially blinatumomab) vs. CAR T and the best sequence in which to use them.

A post-hoc analysis of the ZUMA-3 study (*n* = 78) reported high CR rates with brexucabtagene autoleucel across all subgroups of prior therapy [191]. Composite CR rates were lower for patients who had received prior blinatumomab (63% vs. 83%) or InO (77% vs. 59%) compared to those who had not. Similarly, the median OS was shorter with prior blinatumomab (16 vs. 47 months) or InO (9 vs. 47 months) use. Given the retrospective nature of this analysis, confounding may account for some of these differences.

The recent and timely FELIX study provides a more contemporary view of CAR T cell therapy for R/R B-ALL. The patient population (*n* = 153 including 36 Ph-positive cases) was older than in ELIANA or ZUMA-3, with a median age of 47 years (range 20–81 years), and most patients had previously received blinatumomab (42%), InO (31%), or both (17%). For patients who received an infusion of the CAR T cell product (*n* = 127), the combined response rate was 78% with true CR achieved at 57%. Median EFS and OS were 12 and 16 months, respectively. Among the 99 patients who responded, 18 (18%) subsequently underwent allogeneic HCT. However, there was no significant difference in EFS or OS between those who were and were not transplanted.

Although the FELIX study suggests that CD19-targeted CAR T cell therapy remains efficacious after blinatumomab and/or InO, no prospective trial has directly compared these modalities. Thus, the optimal sequencing of immunotherapies and allogeneic HCT remains to be determined. Moreover, the cost, logistics, and toxicity of CAR T cell therapy have limited its widespread use—especially in medically underserved regions around the globe. Thus, in practice, CAR T cell therapies are still often reserved for fit patients who have relapsed after prior blinatumomab, InO, and/or allogeneic HCT [5]. As CAR T cell therapies become more globally accessible and affordable, potentially through decentralized manufacturing and regulatory optimization, their utilization is expected to expand with increased incorporation into earlier lines of treatment and MRD eradication strategies.

## 7. Conclusions

Immunotherapies such as blinatumomab and InO have dramatically improved survival outcomes for patients with B-ALL. This is, in part, due to the elimination of MRD to provide deep and durable responses. Moreover, they have allowed the intensity of chemotherapy backbones to be reduced, making multiagent therapy more tolerable for broader groups of patients, particularly older adults with other comorbidities. In the case of Ph-positive B-ALL, blinatumomab has even offered an approach to eliminate chemotherapy entirely when used alongside a TKI.

Despite these remarkable advances, several challenges remain. Whereas morphologic response and count recovery (i.e., CR) rates have historically been the main measure of treatment response, MRD has since emerged as a far more sensitive and reliable predictor of long-term disease-free survival. With contemporary induction regimens now offering CR rates >90% for most patients, the comparison of MRD outcomes (in addition to RFS and OS) has become paramount. With this also comes the need to establish standardized techniques for the sensitive and reliable detection of very low levels of MRD [192]. Further, the best course of action for patients with MRD persistence or MRD recurrence after multiagent therapy remains to be determined, and future targeted therapies will need to be evaluated in this setting as opposed to waiting until frank hematologic relapse [193].

The optimal sequence of immunotherapies also remains to be determined. With blinatumomab gaining FDA and EMA approval in the frontline, InO has been increasingly used for relapsed or refractory disease, especially in patients not eligible for HCT. Similarly, the optimal timing of HCT is unclear, especially when CAR T cell therapy is available. That is, whether HCT should be performed early with CAR T relegated to post-HCT relapse or vice versa remains to be determined. Likewise, whether prior receipt of CD19-targeted therapies, such as blinatumomab, impairs the efficacy of CD19-targeted CAR T cells in later lines remains to be determined, although data from pooled pediatric cohorts suggest this remains feasible [194,195].

Finally, the heterogeneity of clinical trial protocols, particularly with the chemotherapy backbone (when used), limits generalizability and confounds the already periled practice of cross-trial comparisons. Although adults have historically benefitted from the use of pediatric-inspired chemotherapy regimens, the field would benefit from identifying more uniform protocols on which investigational agents can be tested, as well as standardized endpoints that can be used for comparison [196].

Ultimately, immunotherapies have established themselves as new standards of care in adult B-ALL. Future therapies will continue to build upon these standards, with particular emphasis on maintaining the high initial response rates, improving the depth and durability of remissions, and reducing treatment-related toxicities to further increase access across age groups.

## Figures and Tables

**Table 1:** Trials in untreated Philadelphia chromosome-negative B-ALL.

NCT	Study/Group	Phase	Author	Year	Population	Induction Regimen	N	Age (Years)	CR (%)	CRD/RFS/EFS (%)	OS (%)	Follow Up
**Anti-CD20 antibodies**												
NCT00671658	MDACC	2	Thomas [36]	2010	CD20+	Rituximab + modified hyper-CVAD	24	53 (19–83)	100	54	65	3 y
NCT00327678	GRAALL-2005	3	Maury [37]	2016	CD20+	Rituximab + standard-of-care chemotherapy	105	40 (25–52)	92	55	61	4 y
NCT01085617	UKALL 14	3	Marks [38]	2022	CD20+/−	Rituximab + standard-of-care chemotherapy	289	46 (23–65)	95	51	NR	3 y
NCT01363128	MDACC	2	Jabbour [39]	2020	CD20+	Ofatumumab + hyper-CVAD	67	41 (32–50)	98	59	68	4 y
**Blinatumomab**												
NCT02143414	SWOG 1318	2	Advani [40]	2022	Older adults	Blinatumomab	29	75 (66–84)	66	66	37	3 y
NCT03480438	GMALL BOLD	2	Goekbuget [41]	2023	Older adults	Blinatumomab + dose-reduced chemotherapy	50	66 (56–76)	76	60	65	3 y
NCT03367299	GIMEMA LAL 2317	2	Chiaretti [42]	2023	Younger adults	Blinatumomab + standard-of-care chemotherapy	149	41 (18–65)	88	66	71	3 y
**Inotuzumab ozogamicin**												
NCT01371630	MDACC	2	Kantarjian [43]	2018	Older adults	InO + dose-reduced chemotherapy	52	68 (64–72)	85	49	56	3 y
Not reported	MDACC	2	Jabbour [44]	2019	Older adults	InO + mini-hyper-CVD ± blinatumomab	58	68 (60–81)	98	49	54	3 y
NCT03249870	EWALL-INO	2	Chevallier [45]	2022	Older adults	InO + dose-reduced chemotherapy	131	68 (55–84)	90	50	54	2 y
NCT03460522	INITIAL-1	2	Stelljes [46]	2024	Older adults	InO + dexamethasone	43	64 (56–80)	100	55	73	3 y
NCT0315069	A041501	3	DeAngelo [47]	2024	AYA	InO + CALGB 10403 chemotherapy	273	27 (18–39)	87	69	79	3 y
**Inotuzumab ozogamicin and blinatumomab**												
NCT01371630	MDACC	2	Jabbour [48]	2023	Older adults	Mini-hyper-CVD + InO + blinatumomab	80	68 (63–72)	89	44	46	5 y
NCT03739814	A041703	2	Wieduwilt [49]	2023	Older adults	InO + blinatumomab	33	71 (60–84)	97	75	84	1 y

Age is presented as median (range). Abbreviations: AYA, adolescents and young adults; CR, complete remission; CRD, complete remission duration; EFS, event-free survival; hyper-CVAD, hyper-fractionated cyclophosphamide, vincristine, doxorubicin, and dexamethasone; InO, inotuzumab ozogamicin; N, number of patients; NCT, national clinical trial; OS, overall survival; RFS, relapse-free survival.

**Table 2:** Trials in MRD-positive Philadelphia chromosome-negative B-ALL.

NCT	Study/Group	Phase	Author	Year	Population	MRD-Adapted Regimen	N	Age (Years)	MRD- CR (%)	CRD/RFS/EFS (%)	OS (%)	Follow Up
**Blinatumomab**												
NCT00560794	GMALL	2	Gokbuget [60]	2017	MRD-positive	Blinatumomab	21	47 (20–77)	80	50	NR	5 y
NCT01207388	BLAST	2	Gokbuget [61]	2020	MRD-positive	Blinatumomab	113	45 (18–76)	78	34	43	5 y
NCT02458014	MDACC	2	Jabbour [62]	2022	MRD-positive	Blinatumomab	37	43 (2–84)	73	63	67	3 y
**Inotuzumab ozogamicin**												
NCT03441061	MDACC	2	Jabbour [63]	2024	MRD-positive	InO	26	46 (19–70)	69	54	60	2 y

Age is presented as median (range). Abbreviations: CR, complete remission; CRD, complete remission duration; EFS, event-free survival; InO, inotuzumab ozogamicin; MRD, measurable residual disease; N, number of patients; NCT, national clinical trial; NR, not reported; OS, overall survival; RFS, relapse-free survival.

**Table 3:** Trials of post-induction consolidation therapy in Philadelphia chromosome-negative B-ALL.

NCT	Study/Group	Phase	Author	Year	Population	Consolidation Regimen	N	Age (Years)	MRD- CR (%)	CRD/RFS/EFS (%)	OS (%)	Follow Up
**Blinatumomab**												
NCT02877303	MDACC	2	Jabbour [68]	2022	ND	Blinatumomab	38	37 (29–45)	100	73	81	3 y
NCT03709719	GRAALL-QUEST	2	Boissel [69]	2022	ND	Blinatumomab	94	34 (18–59)	72	72	79	2.5 y
NCT02003222	E1910	3	Litzow [70]	2024	ND; MRDnegative	Blinatumomab ± chemotherapy	112	52 (30–69)	NA	80	85	3 y

Age is presented as median (range). Abbreviations: CR, complete remission; CRD, complete remission duration; EFS, event-free survival; MRD, measurable residual disease; N, number of patients; NCT, national clinical trial; ND, newly diagnosed; OS, overall survival; RFS, relapse-free survival.

**Table 4:** Trials in relapsed or refractory Philadelphia chromosome-negative B-ALL.

NCT	Study/Group	Phase	Author	Year	Population	Salvage Regimen	N	Age (Years)	CR (%)	Median RFS (mo)	Median OS (mo)
**Epratuzumab**											
NCT00945815	SWOG S0910	2	Advani [72]	2014	CD22+	Clofarabine, cytarabine, and epratuzumab	31	41 (21–69)	52	NR	5
NCT01219816	CHEPRALL	2	Chevallier [73]	2017	CD22+	Hyper-CVAD + epratuzumab	30	35 (21–59)	33	NR	3
**Blinatumomab**											
NCT01209286	German	2	Topp [74]	2014	CD19+	Blinatumomab	36	32 (18–77)	69	8	10
NCT01466179	Multicenter	2	Topp [75]	2015	CD19+	Blinatumomab	189	39 (18–79)	43	6	6
NCT02013167	TOWER	3	Kantarjian [76]	2017	CD19+	Blinatumomab	271	40 (18–80)	44	7	8
NCT04521231	Multicenter	1b	Jabbour [77]	2024	CD19+	Blinatumomab (subcutaneous)	27	52 (19–78)	89	NR	NR
**Inotuzumab ozogamicin**											
NCT01134575	MDACC	2	Kantarjian [78]	2013	CD22+	InO	90	36 (6–80)	59	4	6
NCT01564784	INO-VATE ALL	3	Kantarjian [58]	2016	CD22+	InO	109	47 (18–78)	81	5	8
NCT01371630	MDACC	2	Jabbour [79]	2018	CD22+	Mini-hyper-CVD + InO	59	35 (18–87)	59	8	11
NCT03991884	UW	1	Kopmar [80]	2024	CD22+	DA-EPOCH + InO	24	46 (28–76)	84	10	17
**Inotuzumab ozogamicin and blinatumomab**											
NCT01371630	MDACC	2	Kantarjian [81]	2023	CD22+	Mini-hyper-CVD + InO + blinatumomab	43	42 (18–79)	67	NR	37
NCT01371630	MDACC	2	Short [82]	2024	CD22+	Mini-hyper-CVD + InO + blinatumomab	8	24 (6–61)	63	NR	6

Age is presented as median (range). Abbreviations: CR, complete remission; DA-EPOCH, dose-adjusted etoposide, prednisone, vincristine, cyclophosphamide, and doxorubicin; hyper-CVAD, hyper-fractionated cyclophosphamide, vincristine, doxorubicin, and dexamethasone; InO, inotuzumab ozogamicin; Mo, months; N, number of patients; NCT, national clinical trial; NR, not reported; OS, overall survival; RFS, relapse-free survival.

**Table 5:** Trials in untreated Philadelphia chromosome-positive B-ALL.

NCT	Study/Group	Phase	Author	Year	Population	Regimen	N	Age (Years)	CR (%)	CRD/RFS/EFS (%)	OS (%)	Follow Up
**Anti-CD20 antibodies**												
NCT01429610	Korean ALL-WG	2	Baek [109]	2023	Ph-positive	Rituximab + standard-of-care chemotherapy	32	53 (18–71)	100	52	52	4 y
**Blinatumomab**												
NCT02744768	D-ALBA	2	Foa [110]	2024	Ph-positive	Dasatinib/dexamethasone followed by blinatumomab	63	54 (24–82)	98	76	81	4.5 y
NCT02143414	SWOG 1318	2	Advani [111]	2023	Older adults, Ph-positive	Dasatinib/prednisone followed by blinatumomab	24	73 (65–87)	92	77	87	3 y
NCT03263572	MDACC	2	Jabbour [112]	2023	Ph-positive	Blinatumomab + ponatinib	40	57 (38–72)	93	95	95	1 y

Age is presented as median (range). Abbreviations: CR, complete remission; CRD, complete remission duration; EFS, event-free survival; hyper-CVAD; N, number of patients; NCT, national clinical trial; OS, overall survival; Ph, Philadelphia chromosome; RFS, relapse-free survival.

**Table 6:** Trials in relapsed or refractory Philadelphia chromosome-positive B-ALL.

NCT	Study/Group	Phase	Author	Year	Population	Regimen	N	Age (Years)	CR (%)	Median RFS (mo)	Median OS (mo)
**Blinatumomab**											
NCT02000427	ALCANTARA	2	Martinelli [119]	2021	Ph-positive	Blinatumomab	45	55 (23–78)	36	7	9
NCT03263572	MDACC	2	Jabbour [112]	2023	Ph-positive	Blinatumomab + ponatinib	14	57 (38–72)	92	15	Not reached 79% at 1-year
**Inotuzumab ozogamicin**											
NCT01363297	MDACC	2	Stock [120]	2021	Ph-positive	InO	16	54 (20–75)	56	4	7
NCT01564784	INO-VATE ALL	3	Stock [120]	2021	Ph-positive	InO	22	56 (26–77)	73	4	9
NCT02311998	MDACC	2	Jain [121]	2021	Ph-positive	InO + bosutinib	18	62 (19–74)	83	8	14

Age is presented as median (range). Abbreviations: CR, complete response; InO, inotuzumab ozogamicin; Mo, months; N, number of patients; NCT, national clinical trial; OS, overall survival, Ph, Philadelphia chromosome; RFS, relapse-free survival.

**Table 7:** Investigational therapies in B-ALL.

NCT	Target(s)	Phase	Author	Year	Population	Regimen	N	Age (Years)	CR (%)	RFS (mo)	OS (mo)	Follow Up
**Antibody-based therapies**												
NCT01440179	CD19	2	Kantarjian [134]	2016	R/R B-ALL	Coltuximab ravtansine (SAR3419)	36	50 (18–78)	18	2	NR	Median
NCT02669264	CD19	1	Jain [135]	2020	R/R B-ALL	Loncastuximab tesirine (ADCT-402)	35	55 (20–80)	9	NR	NR	
NCT01891981	CD22	1	Short [136]	2018	R/R B-ALL	Moxetumomab pasudotox (CAT-8015)	16	30 (18–67)	13	1	7	Median
NCT00150944	CD19 and CD22	1	Schindler [137]	2011	R/R B-ALL	Combotox	17	34 (19–72)	0	NR	NR	
NCT02848911	CD19 x CD3	1	Topp [138]	2023	R/R B-ALL	AFM11	17	48 (19–70)	21	NR	2	Median
NCT05579132	CD19 x CD3	1	Wang [139]	2024	R/R B-ALL	CN201	37	NR	32	NR	NR	
NCT01685021	CD19	2	Klisovic [140]	2021	R/R B-ALL	Tafasitamab	22	52 (16–79)	9	1	NR	Median
NCT02767934	PD-1	2	Cassaday [141]	2020	R/R MRD-positive B- or T-ALL	Pembrolizumab	12	52 (22–75)	8	1	13	Median
NCT00724360	HER2	2	Chevallier [142]	2012	R/R B-ALL	Trastuzumab	15	62 (24–80)	0	NR	NR	
**Menin inhibitors**												
NCT04811560	Menin	1	Jabbour [143]	2023	R/R B-ALL	JNJ-75276617	2	63 (19–83)	NR	NR	NR	
**BH3-mimetics**												
NCT03181126	BCL-2 and BCL-XL	1	Pullarkat [144]	2021	R/R B- or T-ALL	Venetoclax + navitoclax + dose-reduced chemotherapy	47	29 (6–72)	28	2	NR	Median
NCT03808610	BCL-2	2	Short [145]	2024	R/R B- or T-ALL	Venetoclax + mini-hyper-CVD	22	46 (20–70)	57	6	7	Median
NCT03576547	BCL-2	2	Short [146]	2021	R/R Ph-positive B-ALL	Venetoclax + ponatinib + dexamethasone (PVD)	9	37 (26–73)	56	100%	72%	1 y
Not applicable	BCL-2	2	Tang [147]	2024	Newly-diagnosed Ph-positive B-ALL	Venetoclax + olverembatinib + dexamethasone (OVD)	10	41 (27–60)	100	100%	100%	7 mo
**HDAC inhibitors**												
NCT00882206	HDAC	2	Burke [148]	2014	R/R B- or T-ALL	Vorinostat + decitabine + chemotherapy	13	16 (3–54)	31	45%	NR	2 mo
ISRCTN 99680465	HDAC	1	Vey [149]	2017	R/R MDS, AML, or ALL	Abexinostat	17	71 (22–77)	0	NR	NR	Median
NCT01132573	HDAC	1	Carraway [150]	2021	Newly-diagnosed or R/R B-ALL	Entinostat + clofarabine ± chemotherapy	28	55 (21–75)	14	NR	NR	
**PI3K-AKT-mTOR inhibitors**												
NCT00968253	mTOR	2	Daver [151]	2015	R/R B- or T-ALL	Everolimus + hyper-CVAD	24	25 (11–64)	6	NR	6	Median
NCT02484430	mTORC1/2	2	Al-Kali [152]	2023	R/R B- or T-ALL	Sapanisertib	15	45 (36–75)	0	NR	2	Median
NCT01396499	PI3K	1	Ragon [153]	2017	R/R AML, ALL, or MPAL	Buparlisib	14	59 (29–86)	0	NR	2	Median
**RAS pathway inhibitors**												
Not reported	Farnesyl-transferase	1	Karp [154]	2001	R/R AML or ALL	Tipifarnib	34	65 (24–77)	6	NR	NR	
NCT00383474	Farnesyl-transferase	1	Lancet [155]	2011	R/R AML or ALL	Tipifarnib + bortezomib	27	70 (47–82)	7	NR	NR	
**Cyclin-dependent kinase inhibitors**												
NCT03472573	CDK4/6	1	Wilde [156]	2023	R/R B-ALL	Palbociclib	7	53 (27–76)	14	NR	NR	
**Proteasome inhibitors**												
NCT02293109	Proteasome	1	Jonas [157]	2021	Newly-diagnosed Ph-negative B- or T-ALL	Carfilzomib + hyper-CVAD	10	38 (23–61)	100	NR	NR	
CTRI/2017/04/008393	Proteasome	2	Jain [158]	2021	Newly-diagnosed Ph-negative CD20+ B-ALL	Bortezomib + rituximab + chemotherapy	35	20 (15–52)	85	79	79	21 mo
**DNA methyltransferase inhibitors**												
NCT00349596	DNMT1	1	Benton [159]	2014	R/R ALL	Decitabine ± hyper-CVAD	30	33 (4–67)	15	15	3	Median

Age is presented as median (range). Abbreviations: AML, acute myeloid leukemia; BCL, B cell lymphoma; CD, cluster of differentiation; CDK4/6, cyclin-dependent kinase 4 and/or 6; CR, complete response; HER2, human epidermal growth factor receptor 2; HDAC, histone deacetylase; hyper-CVAD, hyper-fractionated cyclophosphamide, vincristine, doxorubicin, and dexamethasone; Mo, months; MPAL, multiphenotypic acute leukemia; MRD, measurable residual disease; mTOR, mammalian target of rapamycin; mTORC1/2, mTOR complex 1 and/or 2; N, number of patients; NCT, national clinical trial; NR, not reported; OS, overall survival, PD-1, programmed death receptor 1; Ph, Philadelphia chromosome; PI3K, phosphoinositol-3-kinase; RFS, relapse-free survival; R/R, relapsed or refractory.
